# Can human intelligence safeguard against artificial intelligence? Exploring individual differences in the discernment of human from AI texts

**DOI:** 10.21203/rs.3.rs-4277893/v1

**Published:** 2024-04-29

**Authors:** Jason Chein, Steven Martinez, Alexander Barone

**Affiliations:** Temple University; Temple University; Temple University

## Abstract

Artificial intelligence (AI) models can produce output that closely mimics human-generated content. We examined individual differences in the human ability to differentiate human- from AI-generated texts, exploring relationships with fluid intelligence, executive functioning, empathy, and digital habits. Overall, participants exhibited better than chance text discrimination, with substantial variation across individuals. Fluid intelligence strongly predicted differences in the ability to distinguish human from AI, but executive functioning and empathy did not. Meanwhile, heavier smartphone and social media use predicted misattribution of AI content (mistaking it for human). Determinations about the origin of encountered content also affected sharing preferences, with those who were better able to distinguish human from AI indicating a lower likelihood of sharing AI content online. Word-level differences in linguistic composition of the texts did not meaningfully influence participants’ judgements. These findings inform our understanding of how individual difference factors may shape the course of human interactions with AI-generated information.

## Introduction

Modern generative artificial intelligence (hereafter, gAI) models are aggressively blurring the lines between human and artificial intelligence capabilities. The widening use, and ongoing enhancement of gAI systems, most especially large language models (e.g., ChatGPT^1^, Gemini^2^), has raised concerns about the potential for these tools to amplify cheating and deceitful self-presentation^3–7^ replace the human workforce,^8^ and propagate disinformation.^9,10^ In the scientific arena, AI-generated science abstracts are difficult to spot, and fears are growing that even whole-cloth studies fabricated with gAI could evade detection by the peer-review process.^11–14^ Given these concerns, and the already wide proliferation of information originating from gAIs, it’s vital for us to better understand how human evaluators may engage with such material, and to clarify the conditions and psychological factors that predict when human evaluators may succeed, or fail, to make correct attributions regarding the origins (human or machine) of encountered content.

In the present study, we explore human evaluators’ ability to distinguish content produced by fellow humans from that generated by AI systems, focusing on the central question: Are there individual differences in psychological functioning or experience that can account for variation in the ability to distinguish human-generated from AI-generated content? We also consider whether one’s conclusions about the likely origins (human or AI) of encountered content influence the propensity to share that information with others, and whether there are detectable linguistic qualities in human- and AI-authored texts that might shape decisions about their distinct origins. A limited, but informative, corpus of extant works informs our approach.

## Human evaluation of human vs. gAI outputs

The question of whether humans can tell the difference between materials originating from a human versus artificial source has spurred an already sizeable scientific literature. Work in this space explores human evaluation with an array of different content types, including images, videos, creative outputs, and texts. The findings, unsurprisingly, depend at least in part on the sophistication of the gAI system being used to produce simulated materials, with early studies probing the outputs of less advanced AI models finding that human evaluators could indeed detect the difference.^20,21^ However, in studies using newer gAI models, human evaluators are frequently no better than random chance.^22–26^ Interestingly, judgment accuracy can be near chance even when participants’ self-report high degrees of certainty about the source, a finding that replicates in work with AI-generated faces,^22,23^ videos,^27^ artworks,^28,29^ poetry,^30,31^ and texts.^25,26^ While several factors, including the overall length of a text,^21^ can influence evaluation accuracy and dictate how credible or trustworthy human evaluators find the AI-outputs,^32–34^ it’s clear that modern gAIs are very often able to fool human evaluators.

## Inter-individual variation in discrimination accuracy

A question that has thus far received scant attention is why some individuals are especially good at distinguishing products that have human versus AI origin.^26^ Perhaps these individuals possess specific psychological traits, or have accumulated relevant experience, that enables more effective judgement. Somewhat surprisingly, no study conducted to date has explored the central cognitive skills that determine the success with which someone correctly attributes products to their sources (human vs. AI). Accordingly, as a first foray into this space, the present study examines performance on measures of non-verbal fluid intelligence and executive functioning, with the expectation that those performing better on these tasks may possess core analytic and abstract reasoning skills that advantage their ability to distinguish human from AI.

One of the few studies to consider psychological attributes that could enable more successful discrimination accuracy did so in the context of human- vs. AI-generated poetry.^24^ The authors considered two psychological trait variables: empathy and animism (the tendency to attribute living or human qualities to nonliving entities). Participants were overall just slightly above chance (53%) when judging human poems as human, and were below chance (overattributed human quality) when evaluating AI-generated poems. However, a significant association was found for animism, wherein individuals more inclined to make anthropomorphic attributions were also better at discerning human from AI authored poems. No association was found for empathy, but two empathy subscales approached significance, signaling a possible advantage for individuals who can more capably “read the mind” of the author in human originated works. We pursue this possibility by assessing two aspects of empathy, cognitive empathy and affective empathy.

Relevant domain-specific expertise might also advantage discrimination of human from AI products in that domain, though some studies suggest that expertise doesn’t actually buffer against being deceived by AI-generated products. For example, Art majors are no better than non-majors at discriminating between human and AI-generated poetry,^35^ and college faculty are no better than their students at detecting AI-generated writing samples.^36^ Though one study found that high school teachers were slightly better than their students (70% vs. 62%) at determining which in a pair of essays was human vs. AI, self-reported subject-matter expertise in both groups was unrelated to judgment accuracy.^37^ However, computer science PhD students were found to be substantially better at detecting AI-generated science abstracts than Wikipedia-type articles written for a lay audience,^38^ suggesting that the PhDs’ knowledge of stylistic components of scientific abstracts may have facilitated the distinction for scientifically styled materials. Likewise, in the aforementioned study exploring empathy and animism,^24^ greater prior experience with the tested style of poem (Haiku) was associated with stronger judgment accuracy. Thus, in at least some cases, experience does seem to help, even if expert judgment accuracy is far from ceiling.

In the present study, we used human- and AI-generated text materials from general interest and science-focused topics, with the expectation that lay participants lacking in scientific expertise might perform better with the general interest topics. We also investigated whether individual differences in digital technology habits (e.g., social media use, smartphone checking) might yield differences in human/AI discrimination accuracy. Two competing hypotheses were considered. On the one hand, increased exposure to AI-generated information encountered online might lead to stronger discrimination skills among heavy digital media users. On the other hand, such exposure might acclimatize heavier digital media users to AI-generated materials, and thus weaken discriminability.

## How evaluation influences the propensity to spread information to others

One concern raised by the proliferation of AI-generated information regards its potential to increase the spread of misinformation (e.g., “fake news”), because algorithmic tools make it easy to create and propagate fictive content.^10^ If humans struggle to differentiate human from AI outputs, and cannot rely on subjective experiences (e.g., judgements of credibility) to guide their interactions with AI materials, then these conditions create vulnerability to the spread of false information. However, humans are also known to broadly prefer human-originated materials – a phenomenon dubbed “algorithm aversion”.^39,40^ Thus, human evaluators who are better able to assess the true authorship of materials might be less likely to share and spread AI-fabricated information. Accordingly, we consider whether judgments of human vs. AI origin influence participants’ information sharing preferences, and whether there is a relationship between discrimination skill and self-reported willingness to spread AI-fabricated texts.

## Linguistic qualities of human- and AI-authored texts

To test the success of AI systems, and to safeguard against their misuse, developers are working to build software tools that can distinguish gAI from human outputs. These “automatic detection” tools learn to classify inputs based on extensive training with AI and human exemplars.^15,16^ While automatic detection is improving, these models still make many misattributions, and work best on a limited range of materials.^17^ To characterize the specific linguistic properties that distinguish human from AI texts, Markowitz and colleagues^18^ employed a natural language processing system that codes the words in a text according to a range of linguistic categories. Comparing real human hotel reviews to a set of gAI created “fake” hotel reviews, they observed that gAI texts had a more “analytical” style and exhibited increased use of affective language (stronger positive “emotional tone”). Similar work has shown that AI “smart replies” likewise demonstrate an emotional positivity bias.^19^ Whether these same patterns of linguistic difference generalize to other types of gAI texts, and guide judgements about the origin of these texts, is also considered in the present study.

## The Present Study

To capture a scenario in which human vs. AI evaluation might be made in day-to-day life, we utilized texts taken directly from social media and blog platforms. Turning to the question of human evaluators’ abilities to determine the origins of encountered text materials, we assess overall judgement performance as well as specific psychological factors (fluid intelligence, executive control, empathy) and online experiences that might explain variation in successful evaluation. We hypothesized that group-level performance would be only slightly above random chance levels, with relatively poorer performance when participants had less relevant expertise (science stories). We also hypothesized that individuals possessing strong analytical reasoning and executive processing skills (e.g., higher fluid intelligence and executive control) and a greater inclination toward empathy might be able to make more apt judgments. We further considered whether online experience is associated with evaluation accuracy, testing the competing hypotheses that online experience promotes, or diminishes, the ability to discriminate human and AI texts. We further test the hypothesis that individuals who possess stronger evaluation skills may be less likely to share AI-generated materials. Finally, we probe the specific linguistic characteristics that differentiate human-generated from AI-generated content, seeking to determine whether these characteristics might guide judgments of origin.

## Results

### Can people differentiate between human and AI materials?

We first turned to the question of whether, on average, participants were able to successfully differentiate between the human/AI texts and comments. Statistical comparisons for a Human/AI judgement task were conducted in R,^55^ and specific linear regression models were constructed in the lme4 package. On average, participants were 57% accurate in identifying the origin of human/AI texts, and 78% accurate in identifying the human social media comment, with a wide spread in performance across individuals (Fig. 2). While judgment accuracy was far from perfect, human evaluators were overall significantly better than chance for both texts (t(186) = 10.382, p = .0001) and comments (t(186) = 20.09, p = .0001).

When separated by source, human texts were judged accurately (attributed to human authorship) 61% of the time on average, while AI texts were judged accurately only 53% of the time, indicating a stronger tendency to assign human authorship to AI texts than vice versa. General interest materials were also more accurately evaluated (60%) than scientific content (54%). Overall D’ sensitivity scores indicated modest average discriminability for the texts (M = .40, SD = .56), but as shown in Fig. 3, sensitivity was significantly higher for general interest texts (M = .54, SD = .63) than for science focused texts (M = .21, SD = .60), t(186) = 8.19, p = .0001).

### Do psychological attributes or experience predict the ability to differentiate between human and AI materials?

#### Nonverbal Fluid Intelligence.

Nonverbal fluid intelligence, as measured by the abbreviated RSPM, was strongly associated with the ability to differentiate between human and AI materials (Fig. 4, A/B). RSPM performance (M = 62%, SD = 21%) was highly varied across participants, and linear modeling indicated a highly significant relationship between RSPM accuracy and both D’ sensitivity for texts (b = 0.81, p = .0001) and overall judgment accuracy for comments (b = 0.29, p = .0001). Together, these findings indicate that fluid intelligence, as measured by the RSPM, was strongly associated with the ability to differentiate human from AI materials.

#### Executive Functioning.

Overall Flanker task accuracy was high (*M* = 94%, SD = 11%), but inter-subject variation in accuracy evinced only a trending relationship with Judgment Task D’ for texts (b = 0.69, p = 0.071). There was, however, a significant relationship between Flanker accuracy and overall judgement accuracy for social media comments (b = 0.32, p = 0.01). No significant relationships were found between the Flanker task congruency effect (M = 50.5 ms, SD = 53.3 ms) and either text D’ sensitivity (b = −0.0001, p = 0.88) or overall judgment accuracy for comments (b = −0.0003, p = 0.25). In summary, findings showed that Flanker congruency effects were unrelated to differentiation of human/AI texts, but that Flanker task accuracy was related to enhanced differentiation of human versus AI social media style comments.

#### Empathy.

A potential relationship between empathy and human/AI discrimination accuracy was explored in further testing (Fig. 4, C/D). Overall empathy scores (M = 89.65, SD = 10.47) did not relate to D’ sensitivity (b = −0.001, p = .75) or overall judgment accuracy for comments (b = −0.0004, p = .77). Cognitive empathy (M = 56.63, SD = 7.40) and affective empathy (M = 33.20, SD = 5.66) also exhibited no associations with text D’ (b = −0.003, p = .60; b = 0.0007, p = .93) or judgment accuracy for comments (b = 0.0002 p = .93; b = −0.002, p = .51)

#### Smartphone and Social Media Habits.

Linear regression indicated that MTES composite scores were not significantly related to overall D’ sensitivity for texts (b = − .105, p = .083) or judgment accuracy for comments (b = − .015, p = .46). However, exploratory analyses (Fig. 5) showed that MTES composite score was significantly associated with a greater likelihood to mistake AI texts for human (b = 2.115, p = .014). This finding suggests that increased exposure to mixed AI and human materials online does not enhance one’s ability to differentiate human from AI materials, but rather, may make AI-generated content appear more human-like.

### How does human vs. AI discrimination affect sharing?

Overall, and consistent with “algorithm aversion”, participants showed a significantly stronger self-reported preference for sharing materials that they judged as having a human origin (M = 37.5 SD = 23.8) than those that were judged as having an AI origin (M = 33.3, SD = 23.8); t(186) = 5.65, p = .0001. Interestingly, the preference for sharing materials judged as human but derived from an AI source (M = 38.35, SD = 24.90) was comparable to, and even slightly stronger than, that for actual human material judged as human (M = 36.7, SD = 23.34), suggesting that the attribution, rather than the source, is the driver of sharing preference. As shown in Fig. 6, greater D’ sensitivity on the human/AI Judgment Task was also associated with overall lower average sharing rate (p = .036). This relationship was significant for AI materials (p = .013) and trended in the same direction for human texts (p = .097). This outcome suggests that the ability to detect the AI origin of a social media text can lower the likelihood that an individual will propagate that information into the world.

### Linguistic differences between human and AI materials.

Linguistic composition of the human and AI materials was characterized using the Linguistic Inquiry and Word Count (LIWC) 2022 toolbox.^51^ LIWC is a validated natural language processing tool that codes individual words in a given text according to a set of linguistic categories that index specific psychosocial constructs.^51–54^ To replicate previous reports of heightened analytic and affective features in AI-generated language,^18^ and to explore additional linguistic differences that might exist between human and AI materials, we examined four dimensions coded by LIWC: Analytical Thinking, Emotional Tone, Authenticity, and Clout. In LIWC output, each of these linguistic categories is scaled into a percentile score based on standardized algorithms developed in prior research.

Statistics for LIWC characterization of the linguistic qualities of the human and AI texts are shown in Table 1. A small but significant word count difference was present, wherein AI texts were overall slightly shorter (5 words) than human texts. Despite this word count difference, linear regression contrasts indicated no differences between the human and AI texts for Analytical Thinking, Authenticity, or Clout. The AI texts did, however, produce significantly differentiated Emotional Tone scores, with AI texts, producing greater positive emotional tone than human texts. This pattern held true for both general interest and science-content texts.

To explore whether individuals’ evaluations of AI texts might rely on the single word level characteristics captured by LIWC, additional linear regression models were run to test the relationship between average text judgement accuracy (for a given text) and LIWC composition. For human texts, the only significant association was for LIWC Clout, wherein human texts exhibiting relatively higher Clout scores were more likely to be accurately judged as human (b = .002, p = .042). For AI texts, word count significantly predicted judgement accuracy (b = .004, p = .0018), but no other LIWC dimension produced a significant association. Thus, while relatively longer AI texts were more accurately evaluated, single word-level factors, such as increased positive emotional tone, did not likely drive conclusions about the origin of AI content.

## Discussion

In this study, we explored the human capacity to discriminate between human-generated and AI-generated content, a topic that is already of wide societal interest, and that is likely to become increasingly relevant as AI tools are integrated into the fabric of our day-to-day lives. The findings corroborate observations made in prior research, but also afford several novel insights.

As in several previous studies,^20,21,56^ we found that human evaluation accuracy was significantly, but only slightly, better than chance guessing when materials were considered in isolation. Participant errors reflected a stronger tendency to think AI-generated materials came from a human, rather than the other way around, which also replicates earlier work.^24,27^ Our participants discriminated much more successfully when evaluating content focused on general interest compared to science-focused content. We suspect that this result reflects an advantage for discrimination in content domains that are more familiar to the generalist Prolific recruitment pool, and a relative disadvantage for materials that requires some specialized domain knowledge (e.g., the science stories). This result provides an interesting corollary to prior work showing that science experts made better judgements for scientifically stylized materials relative to content written for a lay audience.^38^ Here, we show that the reverse is also true – a lay audience more accurately judges content that is intended for general readership than content in the scientific domain.

When human and AI-generated comments were considered side-by-side, judgement accuracy was comparatively high (78%). This high accuracy rate for comments was obtained without prior training (cf. ^25,56^) or matching of materials to participants’ domain expertise (cf. ^37,38^). While greater judgement accuracy for comments may reflect salient stylistic cues present in social media comments, the opportunity to make a side-by-side comparison of the human and AI comments is also likely to have advantaged this component of the task. Indeed, average judgement accuracy tends to be relatively higher in studies where a direct comparison between human and gAI materials was supported,^37^ though to our knowledge no previous work has examined evaluation success when the same materials are judge in isolation vs. side-by-side.

Importantly, we observed a very wide range of discrimination skill across individual participants, which allowed us to explore individual difference factors that predicted variation in the ability to differentiate human from AI materials. For the four text categories, the top performing 10% of the sample made accurate judgements better than 70% of the time, and all had D’ scores above 1, indicating that these individuals were able to successfully use information contained in the texts to adjudicate authorship source. Among those who were less able to tell the difference, there was a strong tendency to misattribute human authorship to materials that were actually written by the gAI.

We hypothesized that the analytic and reasoning skills that come with stronger executive functioning and fluid intelligence might empower individuals to distinguish more effectively between human and AI products. Executive functioning, as measured by the Flanker Task, exhibited only a hint of a relationship with discrimination performance, with higher Flanker Accuracy showing a statistically significant association with judgment accuracy for comments, but not the standalone texts. We did, however, find a robust and highly significant relationship between the RSPM measure of fluid intelligence and Judgment Task performance. This relationship persisted for both the texts and comments. Since the two tasks and the RSPM use very distinct material types, and have seemingly disparate analytic demands, this pattern of relationship encourages the conclusion that stronger domain-general analytic and abstract reasoning skills may indeed promote more successful assessment of the authorship of encountered content, This novel finding showcases the importance of central human cognitive abilities in the context of human/AI discernment, and complements the small number of studies showing that expertise in high-functioning or trained groups may facilitate successful discrimination of human and AI information.^37,38^ While previous work with AI-generated artistic expression found hints of a relationship (that did not reach statistical significance) between human evaluation accuracy and trait-level empathy, the present results offer no further corroboration of any such relationship.

Rather than equip individuals with enhanced discrimination prowess, we also found that spending more time online exacerbates the misattribution of gAI outputs as having human origin. Namely, we found a significant association between higher MTES scores, reflecting more intense smartphone and social media use, and the rate of AI misattribution. Though this finding deserves replication, it suggests the possibility that individuals who spend more time interfacing with online content may become inured to the subtle cues that signal origination from gAI. Thus, rather than providing a useful platform for training individuals to discriminate human from AI stories, posts, and comments, habitual social media and smartphone use may actually be worsening the problem.

Another novel finding is the discovery of an association between human/AI discrimination skills and sharing preferences. Similar to previous work on preference for human over algorithmic products,^40,57^ participants in this study indicated a significantly greater preference for sharing materials that they judged (correctly or incorrectly) as having human authorship. Taking a further step, we show that individuals who are better able to distinguish human-generated from AI-generated texts are accordingly less inclined to share AI information. That is, those who were better able to tell the sources apart tended to share less in general but were especially less likely to share actual AI materials. Given broad concern about the widening spread of misinformation, much of which is produced via gAI platforms, this pattern of findings provides at least some hope that the spread of false information could be stemmed through techniques that improve human evaluation.

We also replicate the observation that gAI texts contain more positive emotional language than do human texts.^18,19^ This finding thus appears to capture a general quality of current gAI text outputs. While this difference could, in principle, serve as a guidepost during human evaluation, emotional tone did not predict judgement accuracy, suggesting that this feature was not actually attended to when participants rendered their judgements. We found no effects for single-word text composition along the dimensions of analytical thinking, authenticity, or clout. Indeed, the perhaps most impressive observation is that human and gAI texts were mostly indistinguishable at the single-word content level. Thus, being more proficient at discerning human from AI is likely to reflect the use of linguistic signals that come from the phrasal- or discourse-level of these texts.^25^

While there are several novel and compelling aspects of these data, there are also limitations that deserve consideration. The first, and potentially most important issue is that AI technology is a very fast-moving target. Since data collection was completed for this study, multiple advanced versions of ChatGPT have already been released, and competitor companies (e.g., Google Gemini) have produced other models capable of exceptional human mimicry. We can anticipate that the increasing sophistication and refinement of gAI systems will only deepen the problem of detection, and may shift the landscape of psychological and knowledge skills that facilitate successful discrimination.

Another potential limitation relates to choices made in the construction of study materials, which may have been consequential for the findings. We hoped that the use of social-media focused content would connect the findings to a circumstance that has everyday relevance, since social media interactions constitute one of the major contexts in which AI-generated content is currently encountered in our day-to-day lives. However, since this type of content has not been featured in previous studies of human/AI evaluation, it’s not clear whether the findings generalize to other types of text-based material, or whether similar observations would be made with non-verbal materials such as faces, images, or deep-fake videos. Some prior work has also considered the potentially important distinction between situations in which relatively unadulterated gAI outputs are used without additional human input versus scenarios in which there is intentional human intervention and selection of “optimal” AI outputs.^25,26,28,30^ To maximize the distinguishability of our social-media focused texts, we created and selected AI materials with minimal human intervention. Others have conducted compelling work exploring alternate scenarios where there is a feedback loop between the human and gAI, with the expectation that such interactivity might better characterize the way in which gAI’s are used, or will come to be used, in many real-world settings.^24,26,30^ When there is a “human-in-the-loop” interaction with the gAI, the outputs are likely to be even less differentiable from purely human products, and this may alter, or even obviate, the relevance of specific psychological skills and experience in judgments of origin.

To more fully understand the roles of psychological attributes and experience in supporting judgments about the origins of encountered information, it would also clearly be desirable to collect data from a more robust and wider battery of measures. Future work might consider using an approach that supports construct-level (latent variable) analysis, rather than relying on single measurement instruments for each variable of interest. In the present study we found a strong association with fluid intelligence as measured by the RSPM, but only a weak association with Flanker task performance and no relationship with the empathy questionnaire. Our findings leave open the possibility that other unassessed aspects of executive control (e.g., working memory, planning) and mentalizing skill, along with a host of other unprobed psychological characteristics (e.g., animism, conscientiousness, etc.), might have bearing on one’s skill at distinguishing human from AI.

Ironically, one further concern is the possibility that AI use may have contaminated aspects of the design. Firstly, some of the putatively “human” materials we used could have inadvertently had artificial origins. We tried to mitigate this concern by choosing materials from trusted news media sources and emphasizing a time period that preceded the widening use of AI. The fact that a substantial proportion of participants could successful discriminate the two types of material provides some evidence that the materials were in fact differentiated in origin. As a further check, we also ran a subsample of the texts through automatic AI detection, and found consistent evidence for the separability of the two sets. A second potential issue stems from our use of an online platform for participant recruitment, creating the possibility that some “human” evaluators were actually online bots (computational programs) designed to complete online tasks. This is an issue of increasing concern in online research,^58^ but several aspects of our data alleviate this concern. For instance, online bots would not have produced accurate Flanker performance, above chance discrimination sensitivity, accuracy differences for general interest versus scientific content, or good to high internal consistency on questionnaires.

## Conclusion

We observe that differences in fluid intelligence and online experience account for cross-individual disparity in the ability to discriminate human from AI texts. While there are likely other psychological traits and individual difference factors that similarly explain variance in discrimination skill, furthering our understanding of these factors can place us in a better position to guide human interactions with gAI, and to safeguard against the perils that these tools create. With ever more powerful gAIs hitting the market, detection is going to get harder, and the day may come when human evaluators are wholly unable to tell the difference between original human products and AI-generated imitation. Perhaps by then we will have built more reliable automatic detection tools, enacted regulatory policies (e.g., AI watermarking) that can protect us, and hopefully found ways to leverage findings on individual differences to shape the development of training approaches that enhance human evaluation. For now, human intelligence may just be the best tool we have against artificial intelligence.

## Methods

### Participants

Participants were recruited on Prolific ( www.prolific.com ), a platform that facilitates recruitment for online research studies. Participants were required to be between 18 and 34 years of age, fluent in English, and located in the United States. In total, 203 participants were initially recruited, but nine were excluded from analyses for not completing the full study sequence. The final sample included 194 participants (M age = 28.7 years, SD age = 4.05 years; 68 Female, 4 Non-Binary). All participants provided electronic informed consent, as approved by the Temple University Institutional Review Board. Participants were compensated via electronic payment at an hourly rate of $16 per hour. The mean duration for study completion was 57.2 minutes (SD = 23m).

### Stimuli

Two types of text material were used: online general interest news stories and online scientific news stories. To investigate human detection of potentially AI-generated commentary (e.g., comments written by bots) that may accompany online news, we also investigated the discriminability of human vs. AI comments responding to the primary text items.

### Human- and AI-Generated Texts

Forty-eight human-generated texts were gathered. Thirty general interest news stories were selected from two major social media platforms, Facebook and X. To limit the likelihood that selected materials might have been generated by AI, all items were taken from trusted news organizations (e.g., New York Times, Wall Street Journal) with an emphasis on postings between 2011 and 2014, when AI use was not prevalent. We also avoided major news stories that might have already been familiar to participants. Eighteen scientific news stories were similarly selected from the social media accounts (Facebook and X) of widely disseminated scientific journals (e.g., Science, Nature) and widely read blog websites (or the social media accounts belonging to those websites).

Forty-eight matching AI-generated texts were created using ChatGPT (version 3.5), with 12 exemplars for each of the four text types. Specific prompts were designed to produce AI outputs that paralleled the human texts according to both their central topic and approximate word count. For example, the topic of “heart disease” was taken from one 30-word human-generated headline, and the following prompt was entered into ChatGPT: “Write a 30-word news headline about heart disease”. This basic prompting structure was followed to produce the full set of gAI texts.

### Social Media Comments

A single human-generated social media comment was obtained from the platform that originally hosted each human-generated text, yielding 48 human-generated comments. Specifically, we sought the first substantive comment that explicitly corresponded to the text topic, and that was at least 5 words long. A matching set of 48 AI-generated social media style comments were created using the AI-generated texts to prompt ChatGPT. For example, if matching human and AI-generated texts were about heart disease, and the corresponding human-generated comment on heart disease was 25 words long, then ChatGPT was prompted to “Create a 25-word comment” on the post “in the style of a social media comment”.

### Procedure

**Figure 1:**
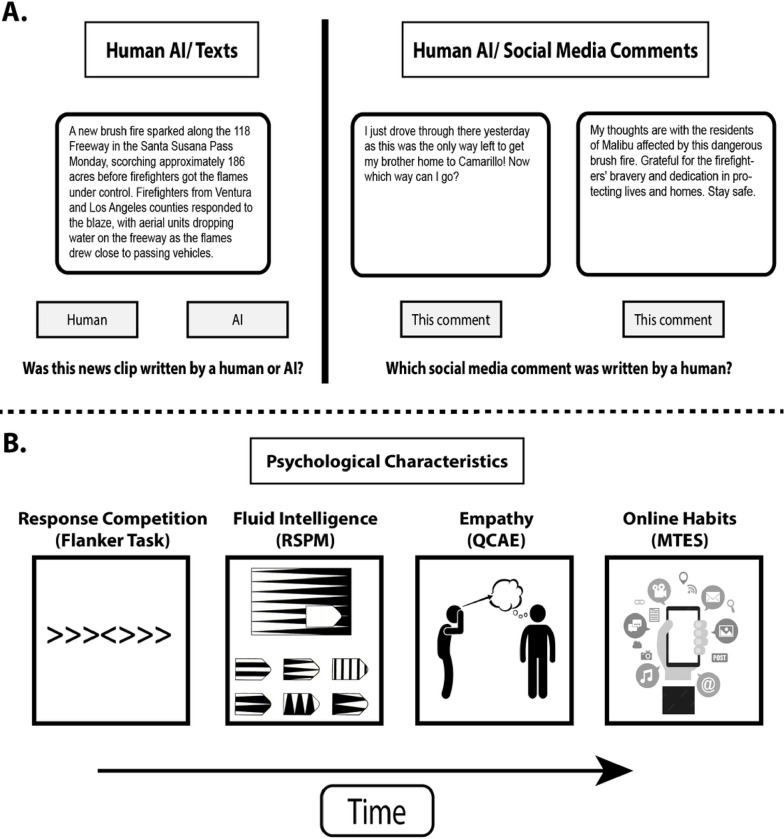
Study design. (A) Online participants first completed a human/AI judgment task in which they had to identify which source, human or AI, had generated the text (left). Two social media comments (right) were then shown, side-by-side, with one written by a human and one by AI. Participants were instructed to select the comment that was written by a human. (B) Participants then completed psychological assessments measuring executive functioning (Flanker task), nonverbal fluid intelligence (Ravens Matrices, RSPM), empathy (Questionnaire for Cognitive and Affective Empathy, QCAE), and smartphone and social media habits (Mobile Technology Engagement Scale, MTES)

**Figure 2:**
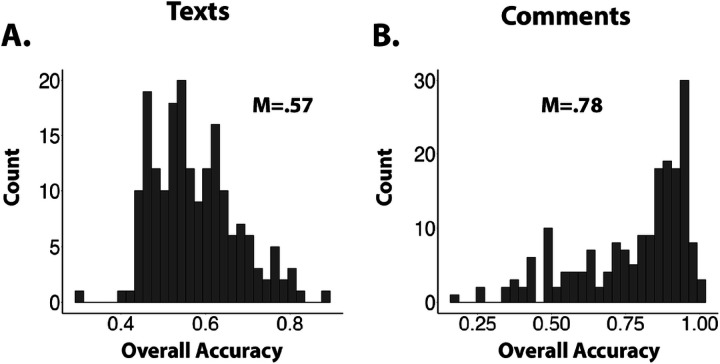
Histograms reflecting the distribution of overall accuracy scores for texts and social media style comments. (A). For texts, participants were 57% accurate on average in determining the correct origin of human/AI materials. (B) For comments, participants were, on average, 78% accurate in determining which of the two comments was written by a human.

**Figure 3:**
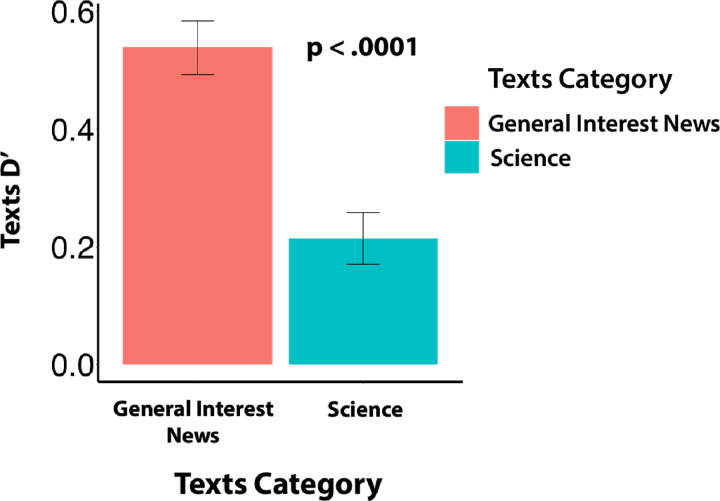
The relationship between text category and D’. Participants were significantly more accurate (paired t-test, two tailed) in determining the origins of general interest news compared to scientific texts. Error bars represent standard error of the mean.

**Figure 4:**
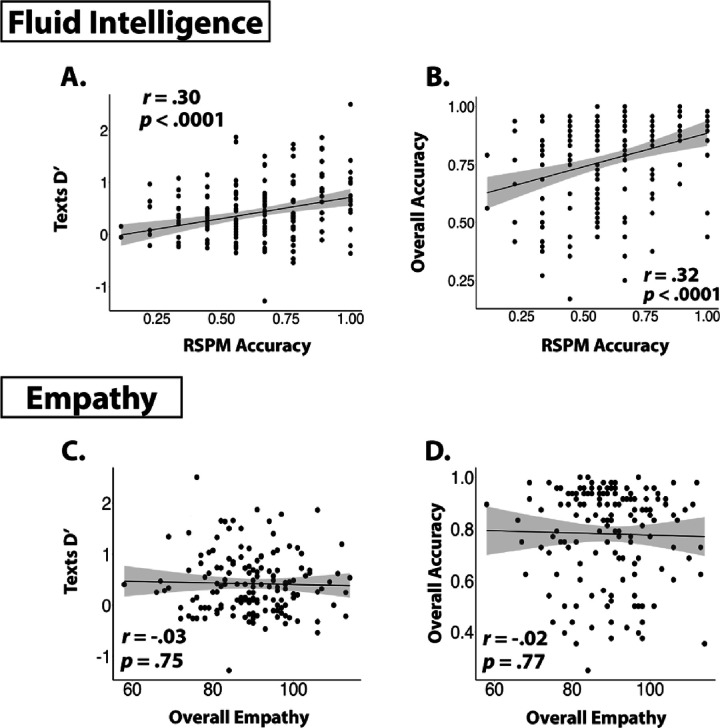
Relationship between discrimination performance and psychological variables. Upper panels show the (A) significant relationship between RSPM and D’ sensitivity for texts, and (B) judgment accuracy for comments. Lower panels show the (C) significant relationship between empathy scale scores and D’ for texts, and (D) the null relationship between empathy scale scores and comment judgement accuracy.

**Figure 5:**
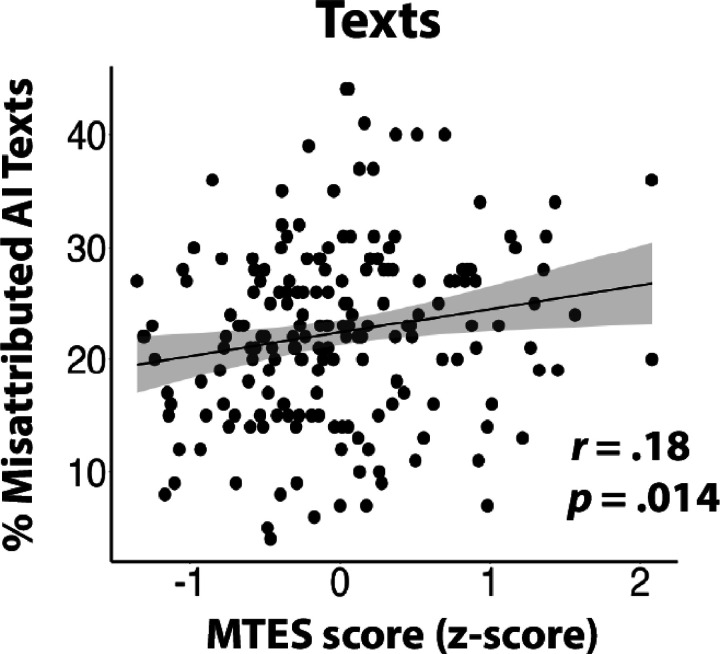
The relationship between smartphone and social media habits and the misattribution of AI texts. Linear regression modelling indicated that those who reported more habitual smartphone and social media use on the MTES were also more likely to mistake AI texts as human.

**Figure 6:**
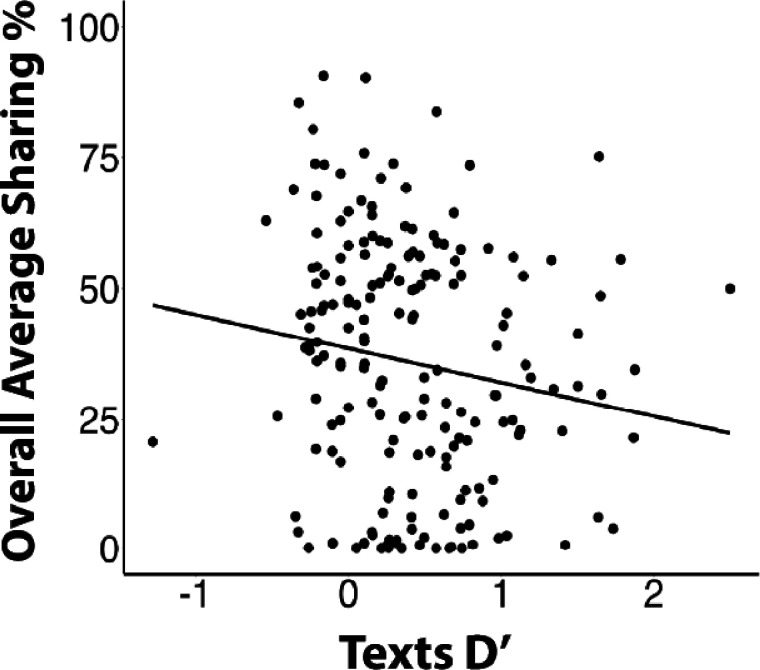
The relationship between text discrimination sensitivity (D’) and overall average sharing preference. Linear regression modelling indicated that those who discriminated better were overall less likely to share.

In an online session, participants completed a series of behavioral tasks, followed by a series of self-report questionnaires. Behavioral tasks assessed the ability to differentiate human- from AI-generated text outputs (Judgment task), executive functioning (Flanker Task), and non-verbal fluid intelligence (Ravens Progressive Matrices). Questionnaires assessed trait-level empathy and smartphone/social media usage habits. These measures were always administered in the order shown in Fig. 1. For all measures, outliers were removed according to task-relevant thresholds.

### Judgement Task

Participants completed a human/AI text judgement task implemented in jsPsych version 7.2.1.^41^ On each trial, a single text was presented, and participants indicated by mouse click whether they believed the text was written by a human or an AI. Choice and response latency data were collected. A total of 96 texts were judged by each participant, with the 48 human and 48 gAI texts presented in random interleaved order. Upon making a choice regarding the origin of each text, two side-by-side social media style comments corresponding to the topic of the preceding text were shown. One of the comments was human-generated, and the other AI-generated, with the left/right placement of each comment type randomly counterbalanced across trials. Participants were instructed to select which of the two comments they thought was written by a human by clicking a button beneath the chosen option. The simultaneous presentation of human- and AI-generated comments was meant to mimic the conditions in which social media comments are typically encountered, with interleaved “real” (human) and algorithmically generated comments shown together in the comments section. Since each of the comments was linked to one of the 48 topics that comprised the originally viewed texts, only 48 *pairs* of social media comments could be presented without replacement. Thus, judgments on these comments occurred following a random selection of 24 human-generated texts and 24 AI-generated texts. To conclude the trial, a slide bar was presented asking participants to indicate how likely they would be to share the original text on social media. Seven participants who selected the same button on over 90% of trials in this task were removed from all analyses involving the Judgment Task.

Two approaches were used to summarize individual performance on the Judgment Task. First, to allow comparison with prior work and ease of interpretability, we calculated judgment accuracy scores as the percentage of texts that were correctly attributed to their human or AI source. Overall judgment accuracy was obtained for the entire series, and separately for each text type. Given our interest in determining the sensitivity with which people can discriminate between human and AI materials, we also calculated D’ signal sensitivity scores, ^42^ using the *psycho* package in R.^43^ The use of a D’ measure protects against extreme response bias and provides a single index of discriminability. D’ scores of 0 reflect chance-level performance, while systematic discrimination is reflected in more extreme positive (accurate discrimination) or negative (inaccurate discrimination) D’ scores.

### Flanker Task

The Eriksen Flanker task^44,45^ assesses selective attention and cognitive control over response competition; key aspects of executive functioning. On each in a series of trials, participants are required to press a keyboard arrow (left or right) corresponding to the direction of a “target” arrow centered on the screen. The target arrow is flanked by three additional, task-irrelevant, arrows (flankers) on each side, which point either in the same direction as the target arrow (“congruent” trials) or the opposing direction (“incongruent” trials). For each trial, the target and flanker arrows remain on the screen for a maximum of 2000ms (terminated on a response), followed by a 300ms inter-trial interval. Participants completed a short practice block consisting of 5 trials, followed by the full experimental task, which consisted of 40 trials (20 congruent, 20 incongruent), presented in randomized order. Accuracy and response time data were collected, and overall performance was operationalized based on average accuracy and the reaction time “congruency effect” (average RT for incongruent trials - average RT for congruent trials). Prior to conducting analyses relating to Flanker Task performance, we removed participants whose congruency effect score was 2.5 standard deviations above or below the average congruency effect score for all subjects, and eliminated individual trials falling above or below 2.5 standard deviations of each individual’s average reaction time. The final sample after outlier removal included 185 participants.

### Raven’s Standard Progressive Matrices Task

Individual differences in nonverbal fluid intelligence were assessed using an abbreviated version of the Raven’s Standard Progressive Matrices (RSPM) task.^46^ The RSPM task requires participants to complete a series of pattern completion problems, and is thought to index abstract, non-verbal, reasoning.^47^ The abbreviated RSPM includes nine problems, shown in increasing order of difficulty. Overall task performance is given by the percentage of correct responses out of the nine problems. The abbreviated version of the RSPM has been shown to reliably predict the total score of the standard 60-item RSPM.^46^

### Questionnaire for Cognitive and Affective Empathy

Trait-level empathy was assessed via the Questionnaire of Cognitive and Affective Empathy^48^ (QCAE). Cognitive empathy, which refers to an individual’s ability to comprehend the internal mental state of another individual, is indexed by summing responses to questions on ‘Perspective Taking’ and ‘Online Simulation’ subscales. Affective empathy, which refers to an individual’s ability to share the emotional experience of another individual, is indexed by summing questions comprising ‘Emotion Contagion’, ‘Proximal Responsivity’, and ‘Peripheral Responsivity’ subscales. Each item on the questionnaire is completed on a 4-point Likert-style scale. ). A subsample of participants (n = 36) did not answer all QCAE questions and were removed prior to analysis. The QCAE exhibits good internal reliability (Cronbach’s alpha ranging from .65 to .85 across subscales),^48^ and demonstrated similar reliability in the present sample for overall empathy (*α* = 0.84), cognitive empathy (*α* = 0.84), and affective empathy (*α* = 0.77

### Mobile Technology Engagement Scale (MTES)

To assess participants’ smartphone and social media habits, we used an updated version of the Mobile Technology Engagement Scale^49,50^ (MTES), which captures three subcomponents of digital media habits: time-based social media use, frequency of online sharing, and phone-checking behaviors. Responses to each question are given on a Likert-style scale, and a composite MTES score is obtained by averaging the z-scores for the three subcomponents. The overall MTES measure has previously yielded acceptable internal reliability (*α* = 0.65–0.68)^49,50^ and in the present sample demonstrated good internal reliability (*α* = 0.73).[1]

## Figures and Tables

**Table 1.** Linguistic differences between human and AI texts.

		Human Texts		AI Texts		Significance Testing
LIWC Category	Description	Mean	SD	Mean	SD	p-value
Word Court	Count of words	50.8	13.6	45.3	10.9	p < .001*
Analytical Thinking	Formal, logical thinking	93.4	8.79	91.6	15.2	0.74
Authenticity	Perceived honesty, genuineness	35.9	31.0	36.1	28.5	0.99
Clout	Confidence, social status, leadership	53.6	10.9	55.6	22.0	0.67
Emotional Tone	Degree of positive or negative tone	37.2	32.6	55.3	38.4	p < .001*

**Note** *SD*= standard deviation.

## Data Availability

Data for the study reported in this manuscript are available on the Open Science Framework via the following link: https://osf.io/4vc8r/?view_only=b53f59c7cf6a4f828727d5c07413f993

## Footnotes

1 Due to a technical error, the first 60 participants completed only one of three MTES questions regarding smartphone checking. Internal reliability was calculated for participants providing complete MTES data. While findings for this measure are reported for the full sample, the same effects also held in the partial sample.
